# Effects of single and repeated shock wave application on the osteogenic differentiation potential of human primary mesenchymal stromal cells and the osteoblastic cell line MG63 *in vitro*

**DOI:** 10.3389/fbioe.2023.1207655

**Published:** 2023-10-12

**Authors:** El-Mustapha Haddouti, Nina Reinhardt, Robert Ossendorff, Christof Burger, Dieter C. Wirtz, Matias de la Fuente, Frank A. Schildberg

**Affiliations:** ^1^ Department of Orthopedics and Trauma Surgery, University Hospital Bonn, Bonn, Germany; ^2^ Chair of Medical Engineering, Helmholtz-Institute for Biomedical Engineering, RWTH Aachen University, Aachen, Germany

**Keywords:** shock wave therapy, shock wave application, *in vitro*, mesenchymal stromal cells, osteogenic differentiation, regenerative medicine, proliferation

## Abstract

**Introduction:** Extracorporeal shock wave therapy is a non-invasive and effective option for treating various musculoskeletal disorders. Recent literature indicates that the parameters for extracorporeal shock wave therapy, such as the optimal intensity, treatment frequency, and localization, are yet to be determined. Studies reporting on the effects of shock wave application on primary mesenchymal stromal cells (MSCs) as well as osteoblastic cell lines *in vitro* are barely available and not standardized.

**Methods:** In this study, we designed a special setup to precisely expose primary MSCs and the osteoblastic cell line MG63 to shock waves and subsequently analyzed the resulting cellular responses using standardized protocols to investigate their viability, proliferation behavior, cytokine secretion, and osteogenic differentiation potential *in vitro.* The shock wave transducer was coupled to a specifically designed water bath containing a 5 mL tube holder. Primary human MSCs and MG63 cells were trypsinated and centrifuged in a 5 mL tube and exposed to single and repeated shock wave application using different intensities and numbers of pulses.

**Results:** Single treatment of MSCs using intensities 5, 10, 15, and 20 and pulse numbers 100, 250, 500, 750, and 1,000 at a constant pulse repetition frequency of 1 Hz resulted in a decreased viability and proliferation of both cell types with an increase in the intensity and number of pulses compared to controls. No significant difference in the osteogenic differentiation was observed at different time intervals in both cell types when a single shock wave application was performed. However, repeated shock wave sessions over three consecutive days of primary MSCs using low intensity levels 0.1 and 1 showed significant osteogenic differentiation 4-fold higher than that of the extracted Alizarin Red S at day 14, whereas MG63 cells showed no significant osteogenic differentiation compared to their corresponding controls. More specifically, repeated shock wave application triggered a significant downregulation of COL1A1, upregulation of RUNX2, and sustained increase of OCN in primary MSCs but not in the cell line MG63 when induced toward the osteogenic differentiation.

**Discussion:** The effects of shock wave application on MSCs make it an effective therapy in regenerative medicine. We established a protocol to analyze a standardized shock wave application on MSCs and were able to determine conditions that enhance the osteogenic differentiation of MSCs *in vitro*.

## Introduction

Shock waves are acoustic waves of short duration that carry energy and can propagate through tissues. They can be mechanical stimulants that cause biological effects in living tissues (Cheng and Wang, 2015; Alvarez, 2022). Extracorporeal shock wave therapy (ESWT) is a non-invasive treatment option for many pathological musculoskeletal conditions like tendon to bone, osteonecrosis of the hip, osteoarthritis, and bone to cartilage (Cheng and Wang, 2015; Alvarez, 2022). Several studies have indicated that ESWT causes the ingrowth of neovascularization in various target tissues, including non-union of long bone fractures (Wang et al., 2009; Wang et al., 2012). The reported biological healing effects of ESWT on tissue regeneration, wound healing, angiogenesis, bone remodeling, and anti-inflammation are thought to be through mechanotransduction processes (Cheng and Wang, 2015). Nevertheless, to date, little is known about the basic mechanism of action of ESWT. The performance of hard tissue regeneration depends on a balance between the osteoinductive stimulant, osteoconductive matrix, and especially osteogenic cell groups (Giannoudis et al., 2007). The bone tissue is under continuous turnover and remodeling, which is a well-regulated biological process during development and fracture healing (Kon et al., 2001; Ambattu et al., 2022). Bone grafts are one of the most commonly transplanted tissues; however, large bone defects caused by trauma and tumors represent an especially challenging clinical problem associated with pain and donor-site morbidity (Pape et al., 2010). Mesenchymal stromal cells (MSCs) have become a field of interest as they provide a possible adjuvant for tissue regeneration. Considering their osteogenic and chondrogenic potential, they are a promising cell population that provides new approaches to regenerating bone tissue (Fayaz et al., 2011). Recent studies have revealed that there are extensive interactions between cells of the bone tissues and cells of the immune system. In addition to their unique property, MSCs not only self-renew and, therefore, contribute to tissue repair and regeneration but also possess immense immunomodulatory capacity (Bernardo and Fibbe, 2013; Eggenhofer et al., 2014). Under inflammatory conditions, MSCs individually respond by homing and integrating into pathological tissues (Rodríguez et al., 1999; Yagi et al., 2010; Ma et al., 2014). These unique immunomodulatory properties have made MSCs a leading cell type of interest having immense potential as a future therapeutic option for the pathophysiological conditions in orthopedics (Faiella and Atoui, 2016; Galipeau et al., 2016). The effects of radial shock wave application (SWA) on osteoblasts were investigated using mice-cultured osteoblasts as a monolayer resulting in an inhibition of osteoblastogenesis (Wang et al., 2002). Interestingly, another study indicated that SWA promotes the differentiation of mice bone marrow stromal cells toward osteoprogenitors (Notarnicola et al., 2012). Moreover, no significant effects on the differentiation potential of the equine adipose tissue-derived mesenchymal stem cells were observed after SWA *in vitro* (Wang et al., 2002). Recently, equine umbilical cord blood MSCs (CB-MSCs) were reported to be responding to SWA *in vitro* by increasing their metabolic activity, but the proliferation was not adversely affected (Raabe et al., 2013). Interestingly, this study showed that SWA on CB-MSCs maintained their multilineage differentiation potential and even increased their potency toward the adipogenic and osteogenic lineages but not the chondrogenic lineage (Raabe et al., 2013). Recently, cell biological effects of mechanical SWA on human bone marrow stromal cells (hBMSCs) cultured as a monolayer were also reported. The study demonstrated that hBMSCs changed their proliferation, migration, and survival after SWA. Furthermore, this study showed that hBMSCs maintained their trilineage differentiation potential, but they did not observe any significant benefit after SWA (Salcedo-Jiménez et al., 2020). Moreover, mechanical stimulation using higher-frequency (1 kHz) vibrations of nanoscale amplitude generated by a bulk piezoelectric actuator has been used to induce the differentiation of MSCs toward the osteogenic lineage but indicating a rather slight increase of osteogenic marker (Suhr et al., 2013; Pemberton et al., 2015). To date, SWA has been reported to be used on MSCs from different sources and species, including mice, equine, and human. Additionally, reported results have been obtained from different types of shock waves and devices and under different experimental conditions, often limited to one or two parameters, which makes the comparison and the reproducibility of the results very difficult. It should be noted that the experimental setup, depending on its design, may influence the results of *in vitro* SWA on cells (Dietz-Laursonn et al., 2016). Moreover, studies reporting on the effects of SWA on MSCs *in vitro* are barely available, not elaborately and systematically generated, and not standardized. There is a need to standardize the applied methods and conditions for SWA when investigating their effects on MSCs *in vitro*. This is of high importance and will enable and advance our understanding of the mode of action and the mechanism of SWA on MSCs *in vitro* and will improve ESWT application in the clinic.

**FIGURE 1:**
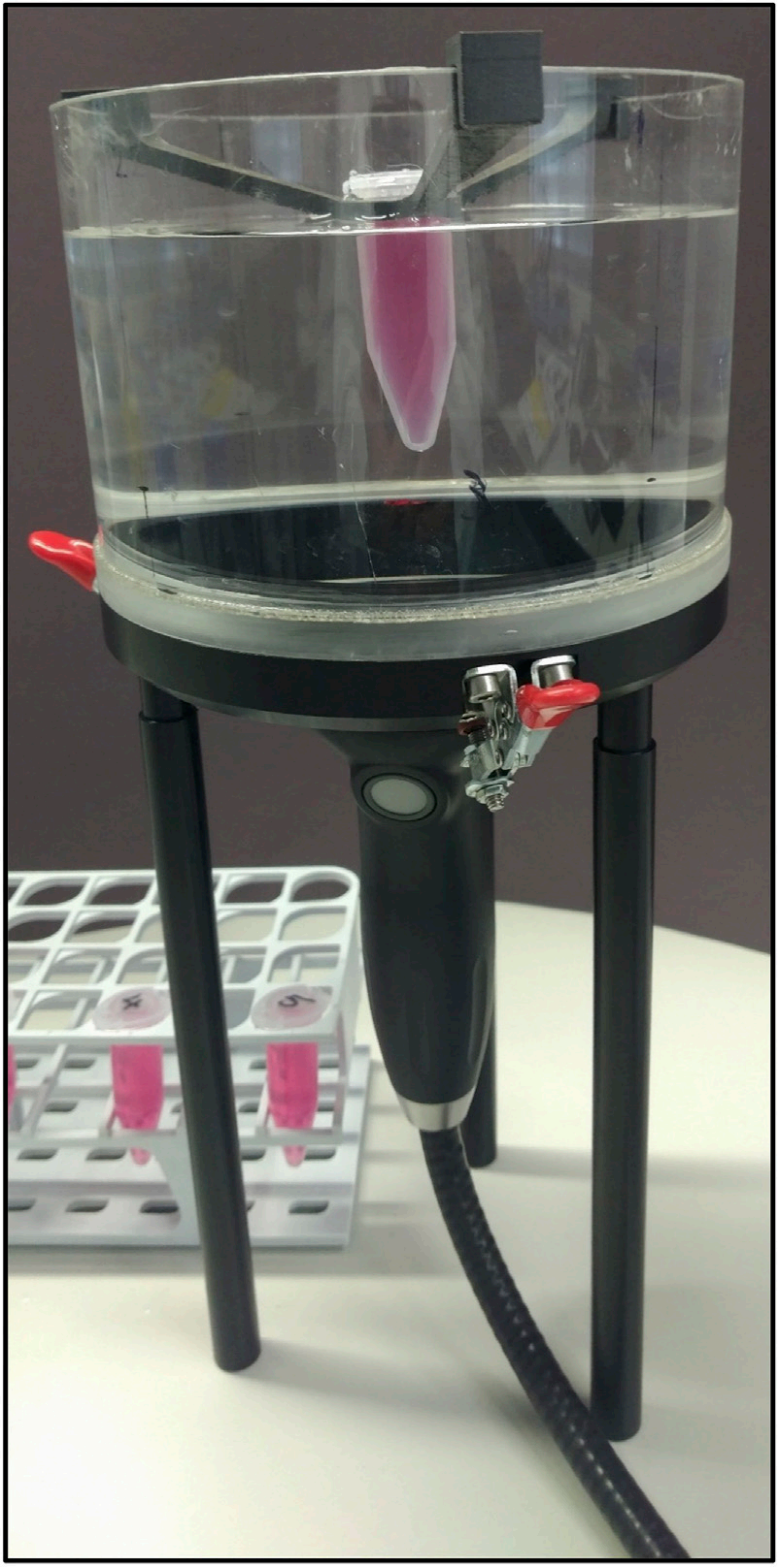
SWA setup. The SWA device, PiezoWave2 with an F10G4 transducer (Richard Wolf GmbH, Germany), was coupled to a water bath containing a 5 mL tube holder. Standardized cultures of human primary MSCs and the cell line MG63 were trypsinated and centrifuged in a 5 mL tube.

In the current study, a commercial ESWT device, PiezoWave2, was used. For this purpose, we designed a special setup to precisely expose MSCs to SWA (Figure 1) and subsequently analyzed the resulting cellular responses. To this end, standardized protocols were systematically established to investigate viability, growth behavior, cytokine secretion, and osteogenic differentiation potential at different time intervals. The aim of this study was to screen different conditions to investigate the responses of the primary MSCs and the cell line MG63 after SWA *in vitro*.

## Materials and methods

### Generation and application of shock waves

The SWA device, PiezoWave2 with an F10G4 transducer (Richard Wolf GmbH, Germany), was coupled to a water bath specifically designed to avoid superposition of the waves due to surface reflections containing a 5 mL tube holder (Figure 1). Air–liquid interfaces in the vicinity of the focal region were avoided by degassing water within the water bath to reduce cavitation effects and kept at a constant temperature (19°C–20°C) at all times. By the actual conceptual design, the pulse repetition frequency was kept constant at 1 Hz, whereas the intensity and the number of pulses were varied. The investigated focused shock wave settings combining different pulse numbers per session and intensities, as well as the respective energy flux density (EFD) determined in water, are indicated in Table 1. According to the manufacturer, the intensity levels correspond to positive peak pressures between 5.8 and 77.7 MPa in water. The lateral focal size ranges from 3.8–1.2 mm (−6 dB zone) and 0.6–9.6 mm (5 MPa zone). Single SWA sessions were performed on both primary MSCs and the cell line MG63, and then, all parameters reported in this study were investigated. Repeated SWA sessions were performed on primary MSCs and the cell line MG63 every 24 h for three consecutive days. Primary MSCs between passages 3 and 5 and the cell line MG63 were trypsinated and counted, and one million cells were centrifuged in a 5 mL tube filled with the culturing medium (Eppendorf, Hamburg, Germany), resulting in a 3D pellet structure. Single and repeated SWA sessions were administered using different intensities and numbers of pulses, keeping the frequency (1 Hz) and the temperature (19°C, 20°C) constant (Table 1). Primary MSCs and the cell line MG63 without SWA were used as controls.

**TABLE 1:** Shock waves were generated at a constant pulse repetition frequency of 1 Hz using the indicated intensities, the number of pulses per session resulting in the depicted EFD, and the indicated number of SWA sessions.

Pulse repetition frequency (Hz)	Intensity	EFD/pulse (mJ/mm^2^)	Number of pulses per session					Number of SWA sessions
1	0.1	0.032	600					3
1	1	0.092	600					3
1	0.1	0.032	600					1
1	1	0.092	600					1
1	5	0.182	100	250	500	750	1,000	1
1	10	0.351	100	250	500	750	1,000	1
1	15	0.582	100	250	500	750	1,000	1
1	20	0.882	100	250	500	750	1,000	1

### Cell culture of primary MSCs and the cell line MG63

Human MSCs were isolated and characterized as previously described (Haddouti et al., 2020a; Haddouti et al., 2020b; Walter et al., 2020). In brief, MSCs were harvested from the femur head after hip replacement. MSCs were isolated through gradient centrifugation (800 × g for 30 min without brake) using Biocoll separating solution (Biochrom AG, Berlin, Germany). MSCs were plated in cell culture flasks (Greiner Bio-One GmbH, Frickenhausen, Germany) with Dulbecco’s modified Eagle’s medium (DMEM) (Gibco by Life Technologies, Darmstadt, Germany) containing 10% serum (Bio&SELL GmbH, Feucht/Nürnberg, Germany), 1% L-glutamine, and 1% penicillin–streptomycin (Biochrom AG). Incubation took place under standard conditions at 37°C in a humidified atmosphere with 5% CO_2_. The culture medium was changed 2–3 times a week. After confluency, MSCs were passaged and stored at −150°C until needed. The MG63 cell line was purchased from the American Type Culture Collection (ATCC) and cultured under the same conditions as MSCs according to the manufacturer’s instructions. The studies involving human participants were reviewed and approved (project IDs: 122/09 and 102/19) and were conducted in accordance with the approved guidelines as well as the Declaration of Helsinki.

### Quantitative estimation of viable cells after SWA

After applying single and repeated shock wave sessions, primary MSCs and the cell line MG63 were seeded in 96-well plates with a standard culture medium at a density of 5,000 cells/well overnight as recommended by the manufacturer (Abcam, Cambridge, United Kingdom). On the following day, cells were incubated with neutral red stain according to the manufacturer’s instructions (Abcam). After 2 h of incubation, the neutral red uptake was measured at OD 540 nm. Three to five MSC donors and three to eight biological replicates per group were analyzed.

### Cell growth properties after SWA

After applying single and repeated shock wave sessions, the proliferation and growth characteristics of primary MSCs and the cell line MG63 were investigated. Cells were plated in 96-well plates at a density of 3,000 cells per well with a standard culture medium to record all phases of cell growth of both cell types. The culture medium was changed every 2 to 3 days. At the indicated time points, cellular optical density (OD) was determined at 570 nm according to the manufacturer’s instructions utilizing the MTT cell proliferation assay (Biotium, Fremont, United States). Three to five MSC donors and three to four biological replicates per group were analyzed.

### Cytokine secretion after SWA

After applying single and repeated shock wave sessions, primary MSCs and the cell line MG63 were counted and seeded at a density of 5,000 cells per well in 96-well plates overnight to detect possibly secreted cytokines. Lipopolysaccharide (LPS), which is a major component of the outer membrane of Gram-negative bacteria and acts as the prototypical endotoxin and promotes the secretion of pro-inflammatory cytokines, was used as the positive control. LPS was added to the control cells at a concentration of 10 μg/mL. On the following day, cell-free supernatants were collected and centrifuged (200 x g, 10 min, 4°C), and aliquots were stored at −80°C until needed. Cytokines interleukin 6 (IL-6), interleukin 1 beta (IL-1β), and tumor necrosis factor alpha (TNFα) were determined using an enzyme-linked immunosorbent assay (ELISA) kit (R&D Systems, Wiesbaden, Germany) according to the manufacturer’s instructions, using a microplate ELISA reader (Tecan, Magellan, Germany). Three to five MSC donors and three to six biological replicates per group were analyzed.

### Osteogenic differentiation after SWA

After applying single and repeated shock wave sessions, primary MSCs and the cell line MG63 were seeded at a density of 1 × 10^3^ cells/cm^2^ in 24-well plates (Greiner Bio-One GmbH) and induced toward the osteoblast lineage by using a culture medium supplemented with 0.1 µM dexamethasone, 10 mM β-glycerophosphate disodium salt hydrate, and 50 µM 2-phosphate-L-ascorbic acid trisodium salt (Sigma-Aldrich, Darmstadt, Germany). A culture medium without any osteogenic induction supplement was used as the control. After 3, 7, 14, and 21 days, cells were fixed with 4% formalin (in PBS, pH 7) (Carl Roth GmbH, Karlsruhe, Germany) and stained with 40 mM Alizarin Red S (pH 4.2) (Sigma-Aldrich, Darmstadt, Germany). After taking representative pictures, the Alizarin Red S staining was extracted using 10% (w/v) cetylpyridinium chloride according to the manufacturer’s instructions (Sigma-Aldrich), and the absorbance was measured at 450 nm using a microplate reader. Three to five MSC donors and four to nine biological replicates per group were analyzed.

### Real-time polymerase chain reaction

To analyze the gene expression of common osteoblast markers, primary MSCs and the cell line MG63 were induced toward the osteoblast lineage after repeated and single SWA sessions, and real-time polymerase chain reaction (RT-PCR) was performed as described previously (Haddouti et al., 2020a). In brief, TRIzol reagent (Ambion, Life Technologies, Darmstadt, Germany) and chloroform: isoamyl alcohol (24:1) (PanReac AppliChem, Darmstadt, Germany) were used for mRNA extraction. Then, 1 µg mRNA was reverse transcribed using a Transcriptor First Strand cDNA Synthesis Kit (Roche Diagnostics GmbH, Mannheim, Germany), and RT-PCR was conducted using LightCycler 480 SYBR Green I Master According to the manufacturer’s instructions (Roche Diagnostics GmbH). Amplifications ran at 95°C for denaturation, 60°C for primer annealing, and 72°C for primer extension for 10 s each for 45 cycles. Primer sequences are listed in Table 2. Data analysis was performed using the ddCT method (Livak and Schmittgen, 2001) determined by normalization to glyceraldehyde 3-phosphate dehydrogenase (*GAPDH*) (Wiraja et al., 2016).

**TABLE 2:** RT-PCR. Accession numbers, and primer sequences used for determining the relative gene expression of *GAPDH*, type I collagen (*COL1A1*), osteocalcin (*OCN*), and runt-related transcription factor 2 (*RUNX2*) in primary MSCs and the cell line MG63 during osteogenic differentiation after repeated and single SWA sessions.

Gene	Primer sequence	Accession number
GAPDH	fwd: 5‘CTC​TGC​TCC​TCC​TGT​TCG​AC3‘ rev: 5‘ACC​AAA​TCC​GTT​GAC​TCC​GA3‘	NM_002046.5
COL1A1	fwd: 5‘TGC​TCG​TGG​AAA​TGA​TGG​TG3‘ rev: 5‘CCT​CGC​TTT​CCT​TCC​TCT​CC3‘	NM_000088.3
OCN	fwd: 5‘GAC​TGT​GAC​GAG​TTG​GCT​GA3‘	NM_199173.6
	rev: 5‘CTG​GAG​AGG​AGC​AGA​ACT​GG3‘	
RUNX2	fwd: 5‘GCG​CAT​TCC​TCA​TCC​CAG​TA3‘ rev: 5‘GGC​TCA​GGT​AGG​AGG​GGT​AA3‘	NM_001,024,630.3

### Statistical analysis

Data are expressed as the average ± SD of three to five MSC donors and three to eight biological replicates as indicated. The D´Agostino–Pearson test or graphical analysis was performed to assess normality in all measured values. Statistical analysis was carried out using GraphPad Prism 7 (GraphPad, La Jolla, CA, United States). For data with Gaussian distribution, a two-tailed Student’s *t*-test was selected. Significance levels are marked as *p < 0.05, **p < 0.01, and ***p < 0.001.

## Results

### Viability and growth properties of cells after a single SWA

**FIGURE 2:**
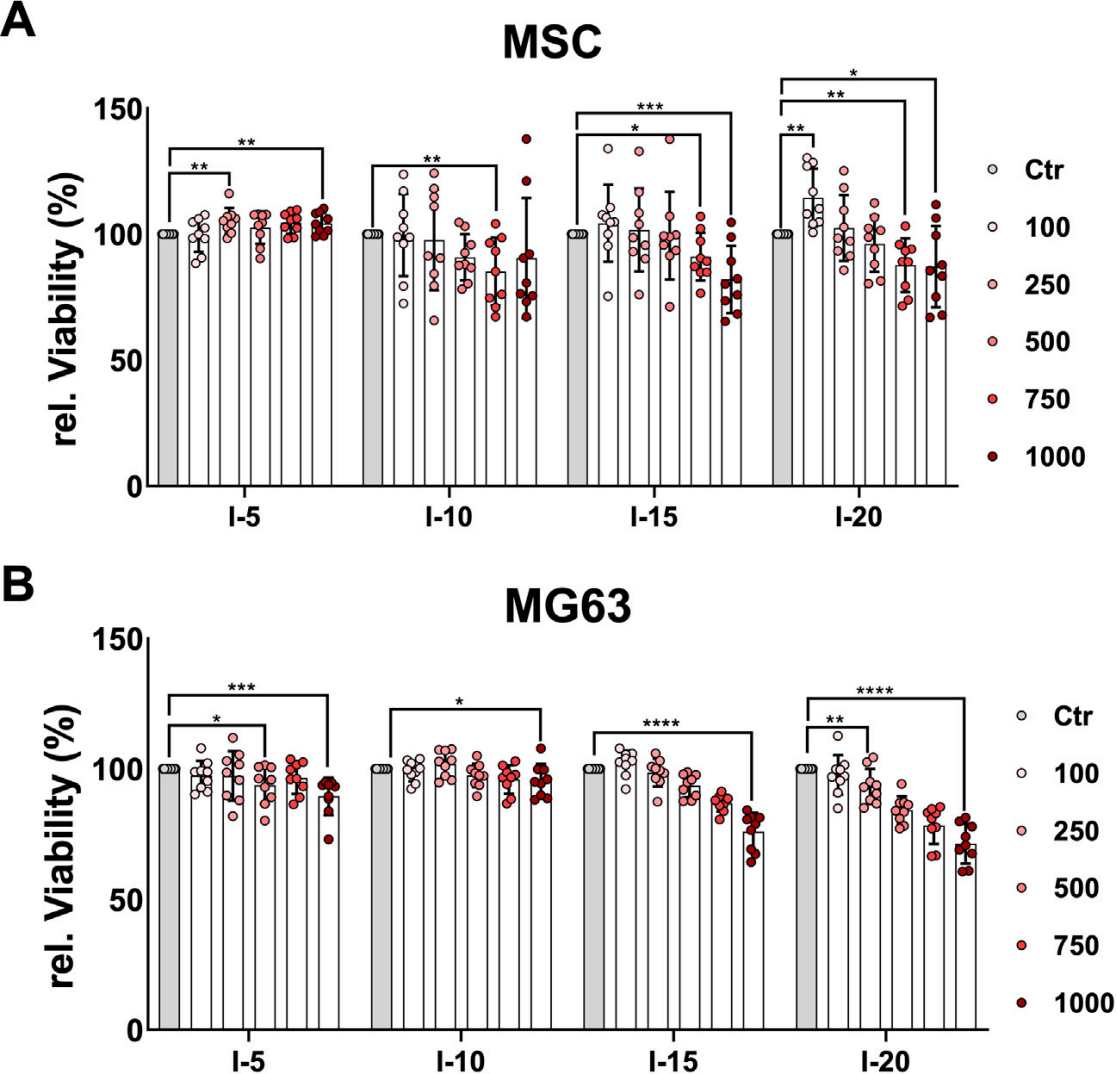
Quantitative estimation of cell viability after a single SWA. Primary MSCs **(A)** and the cell line MG63 **(B)** were stimulated with single sessions of SWA at the indicated intensities (5, 10, 15, and 20) and number of pulses (100, 250, 500, 750, and 1,000) for each intensity. Viable cells normalized to controls (%) were estimated by measuring the neutral red uptake assay on day 1 after SWA at OD 540 nm. Data are expressed as the average ± SD of three to five MSC donors and four to eight biological replicates per group. *p < 0.05, **p < 0.01, and ***p < 0.001, two-tailed Student’s *t*-test.

In order to quantitatively estimate the viability of cells after a single SWA, the neutral red uptake assay was used. The results showed that the viability of MSCs increased significantly when 250, 750, and 1,000 pulses with intensity 5 were applied (Figure 2A), while the MG63 cell line showed a significant decrease in viability compared to the corresponding controls, especially at 1,000 pulses (Figure 2B). When intensities 10, 15, and 20, corresponding to EFDs of 0.351, 0.582, and 0.882 mJ/mm^2^, respectively, were applied, both the primary MSCs and the cell line MG63 demonstrated a significant decrease in viability with an increase in the number of pulses compared to the corresponding controls (Figure 2).

**FIGURE 3:**
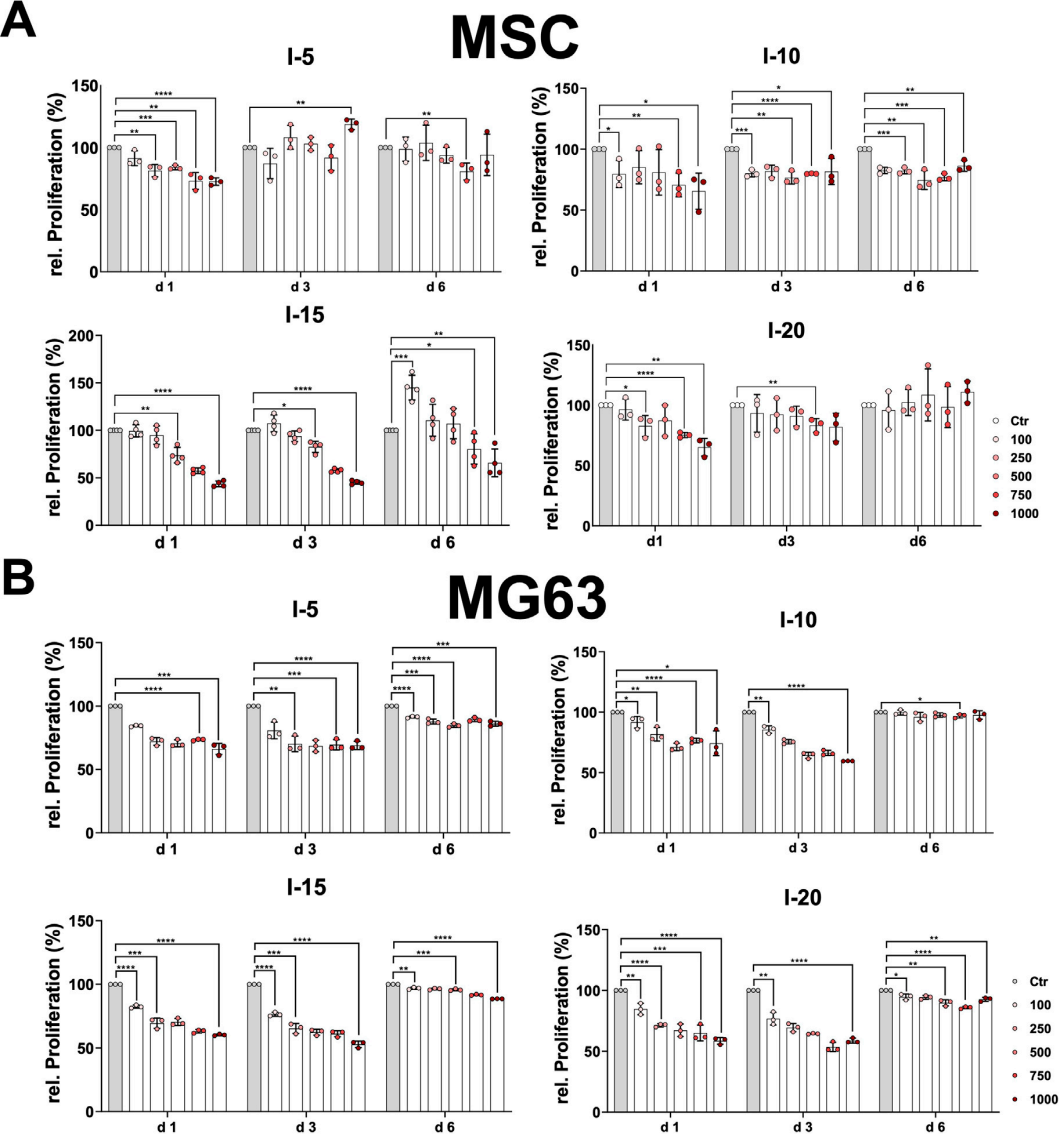
Relative proliferation after a single SWA. Primary MSCs **(A)** and the cell line MG63 **(B)** were stimulated with single sessions of SWA at the indicated intensities (5, 10, 15, and 20) and number of pulses (100, 250, 500, 750, and 1,000) for each intensity. The relative proliferation of cells normalized to the corresponding controls (%) was estimated by measuring the OD 570 nm at the indicated time intervals (days 1, 3, and 6) using the MTT cell proliferation assay. Data are expressed as the average ± SD of three to five MSC donors and three to four biological replicates per group. *p < 0.05, **p < 0.01, and ***p < 0.001, two-tailed Student’s *t*-test.

Moreover, we investigated the growth behavior and proliferation of primary MSCs and the cell line MG63 after a single SWA. To this end, the MTT proliferation assay was analyzed at three time points, namely, on day 1, day 3, and day 6. The growth pattern of the primary MSCs on day 1 after a single SWA decreased significantly with an increase in the number of pulses at all four intensities (5, 10, 15, and 20) applied compared to the corresponding controls (Figure 3A). MSCs stimulated with intensity 5, corresponding to an EFD of 0.182 mJ/mm^2^, showed a clear recovery already on day 3 at all pulse numbers applied, whereas MSCs stimulated with intensity 20 corresponding to an EFD of 0.882 mJ/mm^2^ recovered on day 6. In contrast, MSCs stimulated with intensities 10 and 15, corresponding to EFDs of 0.351 and 0.582 mJ/mm^2^, respectively, were still not recovered on day 6 after a single SWA. Interestingly, MSCs stimulated with intensity 15 showed significantly higher growth when 100 pulses were applied compared to the corresponding control (Figure 3A).

The cell line MG63 showed the same growth pattern at day 1 and day 3, indicating a significant decrease with an increase in pulse number at all four intensities applied compared to the corresponding controls (Figure 3B). The growth behavior of the cell line MG63 demonstrated a tendency toward full recovery that manifested in a kind of plateau for all conditions applied (Figure 3B).

### Cytokine secretion after a single SWA

Next, we considered investigating how the application of a single shock wave could affect cytokine secretion in primary MSCs and the MG63 cell line. To this end, the cytokines IL-6, IL-1β, and TNFα were determined after single shock wave sessions at intensities 5, 10, 15, and 20 corresponding to the EFD of 0.182, 0.351, 0.582, and 0.882 mJ/mm^2^, respectively, using the number of pulses 100, 250, 500, 750, and 1,000 for each intensity. The cytokines IL-1β and TNFα were not detectable under all investigated conditions for both primary MSCs and the cell line MG63.

**FIGURE 4:**
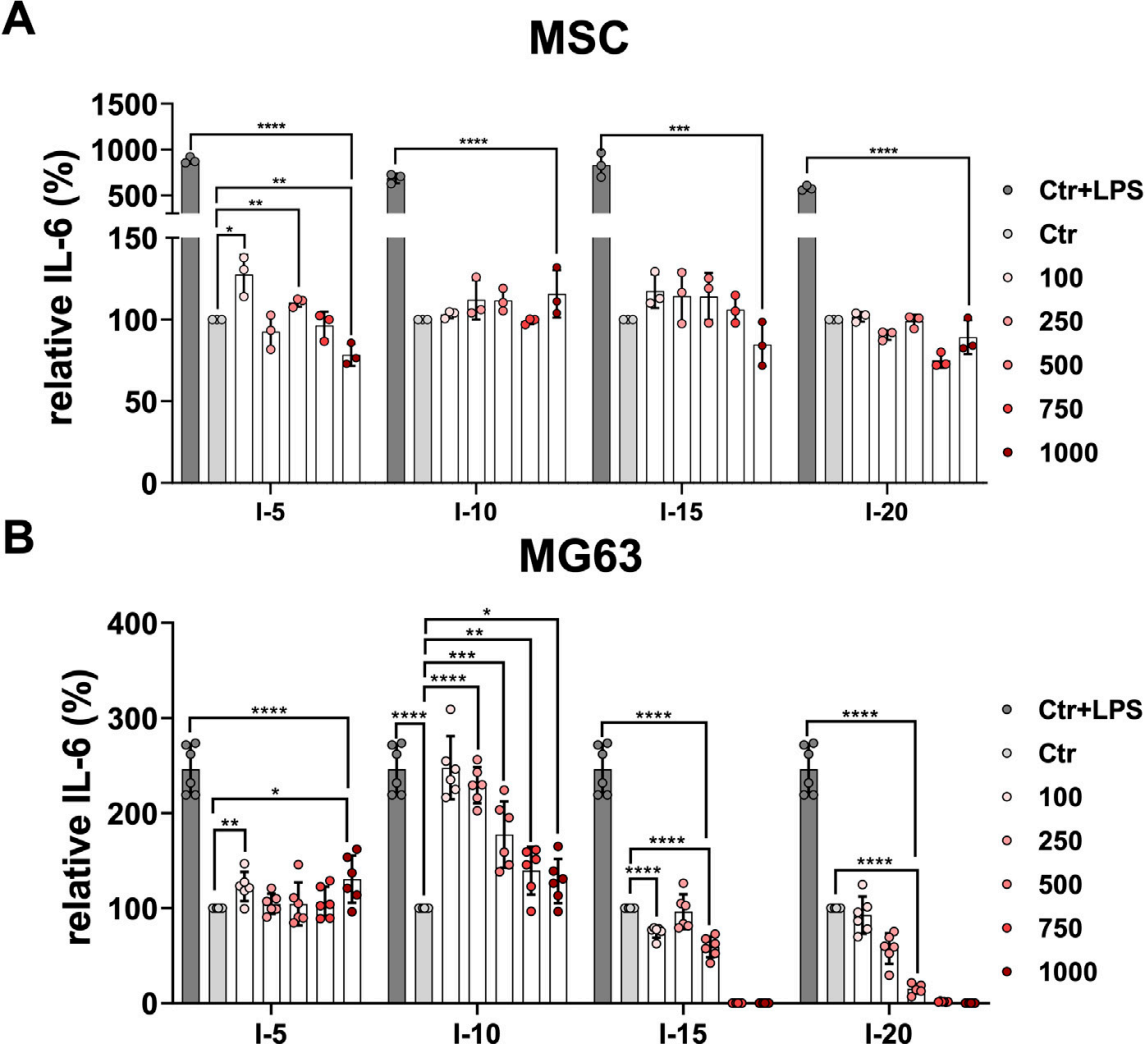
Relative IL-6 secretion after a single SWA. Primary MSCs **(A)** and the cell line MG63 **(B)** were stimulated with single sessions of SWA at indicated intensities (5, 10, 15, and 20) and number of pulses (100, 250, 500, 750, and 1,000) for each intensity. The relative IL-6 secreted was measured with ELISA normalized to the corresponding controls (%). LPS was added to the control cells at a concentration of 10 μg/mL as the positive control. Data are expressed as the average ± SD of three to five MSC donors and three to six biological replicates per group. *p < 0.05, **p < 0.01, and ***p < 0.001, two-tailed Student’s *t*-test.

It has been previously demonstrated that MSCs respond to the inflammatory agent LPS (Kurte et al., 2020). The primary MSCs indicated a 10-fold increase of the relative IL-6 when stimulated with LPS used as the positive control compared to non-stimulated controls as well as all single intensities at the number of pulses applied (Figure 4A). Primary MSCs demonstrated a significant difference in the relative secreted cytokine IL-6 when 100 and 500 pulses of intensity 5 corresponding to an EFD of 18.2 and 91 mJ/mm^2^, respectively, were applied compared to the corresponding control (Figure 4A). In contrast, for intensities 10, 15, and 20, all numbers of pulses applied showed no significant difference in the relative IL-6 compared to their corresponding controls (Figure 4A). Stimulating MG63 with LPS is a well-established inflammatory model (Dai et al., 2021). The cell line MG63 indicated a 2.5-fold increase of the relative IL-6 secreted when stimulated with LPS used as a positive control compared to non-stimulated controls (Figure 4B). The cell line MG63 demonstrated a slightly significant increase of the relative secreted IL-6, especially when 100 pulses and 1,000 pulses of intensity 5 corresponding to an EFD of 18.2 and 182 mJ/mm^2^, respectively, were applied compared to the corresponding control (Figure 4B). Surprisingly, the amount of the relative IL-6 secreted by the cell line MG63 after the intensity 10 application was significantly higher at all pulse numbers applied compared to the corresponding control. Interestingly, 100 and 250 pulses corresponding to an EFD of 35.1 and 87.75 mJ/mm^2^, respectively, showed approximately a 2.5-fold increase compared to the corresponding control and were approximately as high as the corresponding positive control (Figure 4B). After application of intensities 15 and 20, the cell line MG63 showed gradually and significantly decreasing levels of the relative secreted level of IL-6 at the pulses 100 to 500 compared to the corresponding controls. Moreover, the relative IL-6 secreted was not detectable at the pulses 750 and 1,000 when intensities 15 and 20 were applied (Figure 4B).

### Osteogenic differentiation after a single SWA

**FIGURE 5:**
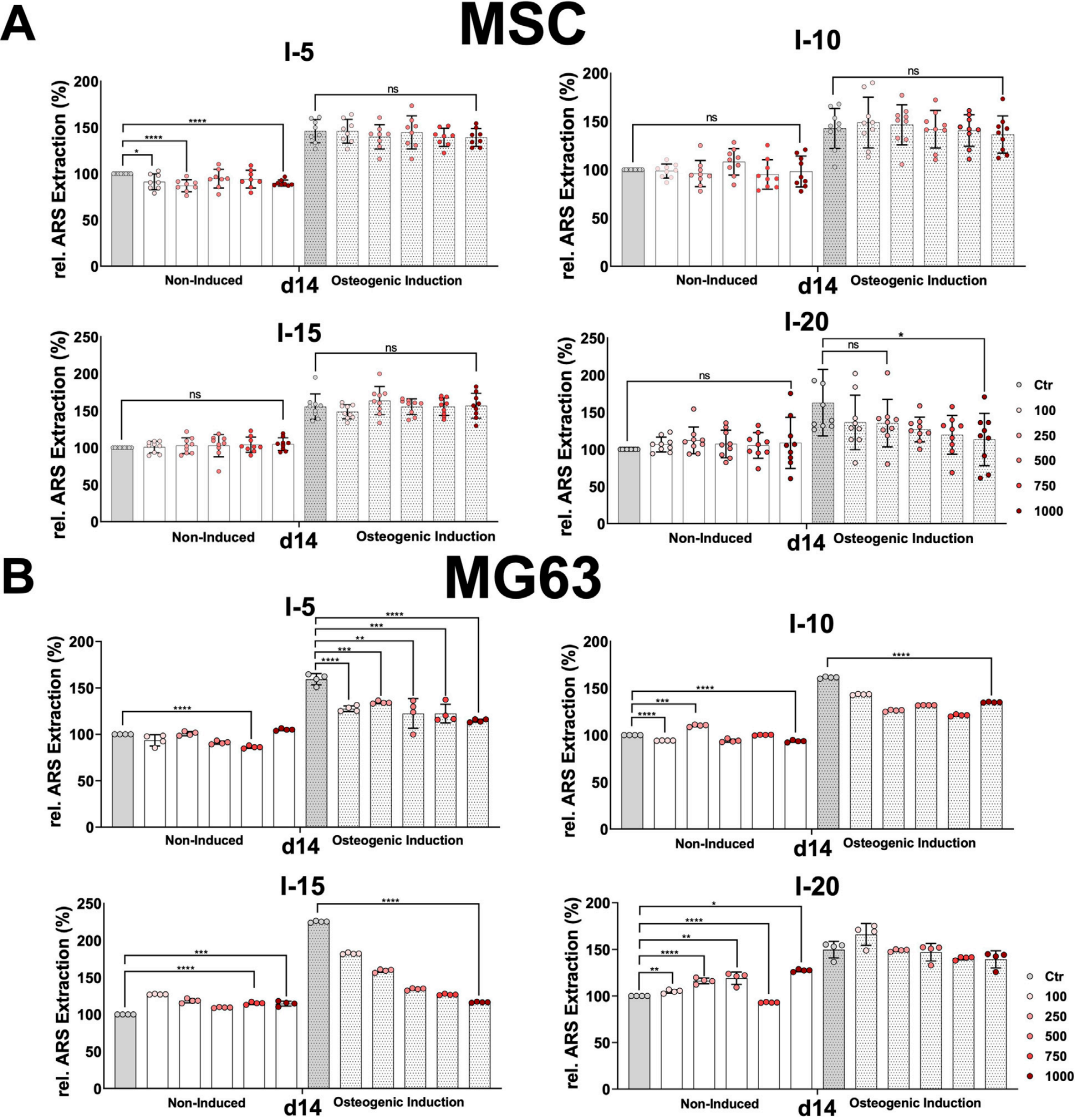
Alizarin Red S extraction from osteogenic differentiated MSCs and the cell line MG63 after a single SWA. Primary MSCs **(A)** and the cell line MG63 **(B)** were stimulated with single sessions of shock wave application at indicated intensities (5, 10, 15, and 20) and number of pulses (100, 250, 500, 750, and 1,000) for each intensity. MSCs and the cell line MG63 were then induced toward the osteogenic lineage for 14 days, and a culture medium without any osteogenic induction supplement was used as the control. The mineralization-specific staining Alizarin Red S was extracted using 10% (w/v) cetylpyridinium chloride, and the absorbance was measured at 450 nm. Data are expressed as the average ± SD of three to five MSC donors and four to nine biological replicates per group. ns: non-significant. *p < 0.05, **p < 0.01, and ***p < 0.001, two-tailed Student’s *t*-test.

Next, we investigated how the application of single shock wave sessions could affect the differentiation toward the osteoblastic lineage of the primary MSCs and the cell line MG63. To this end, both cell types were examined for their mineralization potential during the osteogenic differentiation process at different time intervals via Alizarin Red S staining, which was evaluated histologically as well as through absorbance measurement of extracted Alizarin Red S from stained MSCs and the MG63 cell line (Figure 5).

Histological analysis of Alizarin Red S-stained MSCs and MG63 cells after SWA did not show any significant difference between all intensities and pulses applied compared to their corresponding controls on days 3, 7, 14, and 21 for both cell types. However, Alizarin Red S staining indicated stronger mineralization toward the osteogenic lineage-induced MSCs and MG63 cells compared to non-induced corresponding controls under all conditions applied.

In order to accurately estimate the mineralization potential, Alizarin Red S was extracted from stained MSCs and MG63, and its absorbance was measured under all conditions examined. Nevertheless, primary MSCs and MG63 showed no significant difference in the relative extracted Alizarin Red S, at all conditions and time intervals, during the osteogenic differentiation process for both the non-induced and the osteoblastic lineage-induced samples compared to their corresponding controls after single shock wave sessions. The obtained results of Alizarin Red S extraction from MSCs and the cell line MG63 on day 14 are shown in Figure 5.

For both non-induced and osteogenic lineage-induced MSCs, no significant increase was observed in the mineralization potential under all conditions and all time points investigated compared to their corresponding controls after single shock wave sessions (Figure 5A). A rather significantly decreased mineralization was observed for intensity 5 for non-induced MSCs and intensity 20 for osteogenic-induced MSCs after a single shock wave session (Figure 5A).

This is true also for the cell line MG63 when intensities 5 and 10 at all pulses were applied (Figure 5B). However, when intensities 15 and 20 were applied, non-induced MG63 cells indicated a significant increase in their mineralization potential at all pulses applied compared to their corresponding controls (Figure 5B).

### Repeated SWA

Even though the treatment with ESWT is carried out during repeated sessions, studies on repeated SWA *in vitro* are scarcely available. It is of high relevance to investigate repeated SWA using an *in vitro* cell culture model in order to understand the mechanism of action of shock waves and the benefits and/or harm of repeated ESWT treatment. To this end, the primary MSCs and the cell line MG63 were stimulated with repeated shock wave sessions every 24 h for three consecutive days. In the first step, repeated SWA using intensities 5, 10, 15, and 20 corresponding to EFDs of 0.182, 0.351, 0.582, and 0.882 mJ/mm^2^, respectively, for 600 pulses (10 min), was investigated and led to high death rate on the third day. In the next step, repeated shock wave sessions using low intensities of 0.1 and 1 corresponding to EFDs of 0.032 and 0.092 mJ/mm^2^, respectively, were considered.

To this end, primary MSCs and the cell line MG63 were subjected to repeated SWA with intensities 0.1 and 1 for three consecutive days indicated as (3xI-0.1) and (3xI-1), respectively. Additionally, a single SWA was performed with the same intensities of 0.1 and 1, indicated as (1xI-0.1) and (1xI-1). Cells of both types without any SWA were used as controls.

Viability, growth behavior, and secretion of the cytokines IL-6, IL-1β, and TNFα were also examined after repeated SWA sessions, as mentioned previously, on primary MSCs and the cell line MG63. Remarkably, no significant difference in the relative cell viability and the relative secreted IL-6 was found between most conditions when single and repeated SWA of intensities 0.1 and 1 were applied (Supplementary Figure S1A, C). Interestingly, no significant difference in the relative proliferation of primary MSCs and the cell line MG63 was observed, especially on day 6 (Supplementary Figure S1B).

Furthermore, MSCs and the cell line MG63 indicated no significant difference in the relative viability after single and repeated SWA with intensities of 0.1 and 1, respectively, compared to the corresponding controls (Supplementary Figure S1A). Moreover, the relative proliferation after repeated SWA sessions showed a slight increase for MSCs and a significant decrease for MG63 on day 6 compared to the corresponding controls (Supplementary Figure S1B).

The relative IL-6 secreted when stimulated with LPS used as a positive control was significantly higher compared to non-stimulated controls. The level of the relative IL-6 secreted was slightly decreased in the case of MSCs and non-significantly or only slightly increased in the case of MG63 after repeated SWA compared to the corresponding controls (Supplementary Figure S1C). The cytokines IL-1b, and TNFα were not detectable under all investigated conditions after repeated shock wave sessions for both primary MSCs and the cell line MG63.

### Osteogenic differentiation after repeated SWA

**FIGURE 6:**
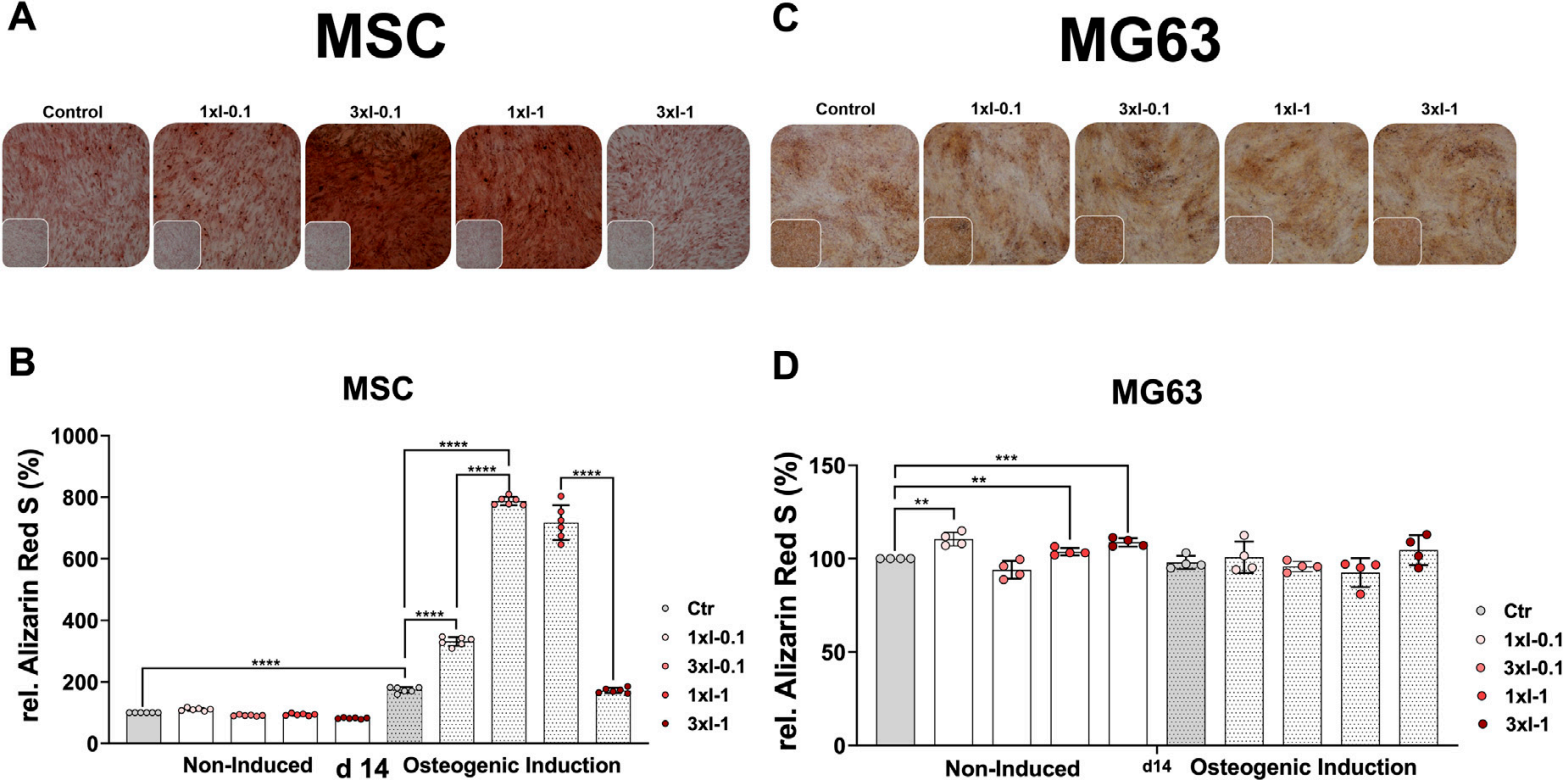
Alizarin Red S staining and extraction of osteogenic differentiated cells after repeated SWA. Primary MSCs (left) and the cell line MG63 (right) were stimulated with repeated intensities of 0.1 (3xI-0.1) and 1 (3xI-1) and single intensities of 0.1 (1xI-0.1) and 1 (1xI-1) shock wave application sessions for 10 min (600 pulses), as indicated. MSCs and MG63 cells were then induced toward the osteogenic lineage for 14 days. A culture medium without any osteogenic induction supplement was used as the control. The mineralization potential was investigated via Alizarin Red S staining. The mineralization-specific staining Alizarin Red S was extracted using 10% (w/v) cetylpyridinium chloride, and the absorbance was measured at 450 nm. (**A, C**) Alizarin Red S staining of primary MSCs and the cell line MG63, magnification ×40. (**B, D**) Alizarin Red S extraction from primary MSCs and the cell line MG63. Data are expressed as the average ± SD of three to five MSC donors and four to six biological replicates per group in parallel to the cell line MG63. *p < 0.05, **p < 0.01, and ***p < 0.001, two-tailed Student’s *t*-test.

Next, we investigated how repeated SWA affects the osteoblastic differentiation of the primary MSCs and the cell line MG63. To this end, both cell types were examined for their mineralization potential during the osteogenic differentiation process at different time intervals after repeated shock wave sessions of 3xI-0.1 and 3xI-1 and single shock wave sessions of 1xI-0.1 and 1xI-1. Cells of both types without any SWA were used as controls. The histological evaluation of Alizarin Red S staining showed strong staining of MSCs stimulated with the repeated intensity of 0.1 (3xI-0.1) and the single intensity of 1 (1xI-1) corresponding to an EFD of 0.032 mJ/mm^2^ for three consecutive days with 600 pulses and an EFD of 0.092 mJ/mm^2^ for one time, respectively, on day 14 during the osteogenic induction (Figure 6A). Moreover, a single session of the intensity of 0.1 (1xI-0.1) on MSCs demonstrated a slight increase of Alizarin Red S staining, whereas the repeated intensity of 1 (3xI-1) showed similar Alizarin Red S staining as the control on day 14 (Figure 6A).

The histological evaluation of the cell line MG63 mineralization potential on day 14 showed no significant increase of Alizarin Red S staining after single and repeated SWA sessions of intensities 0.1 and 1 at 600 pulses compared to the corresponding controls (Figure 6C).

The osteogenic differentiation was additionally evaluated via extraction of the specific Alizarin Red S from stained MSCs and the cell line MG63 to quantify their mineralization potential more accurately. The successful osteogenic differentiation of MSCs was substantiated in a 2-fold increase of the extracted Alizarin Red S in the case of the osteogenic-induced control MSCs compared to the corresponding non-induced control on day 14. Furthermore, shock wave-stimulated MSCs and non-induced MSCs indicated no significant difference in the extracted Alizarin Red S compared to the corresponding control (Figure 6B). Interestingly, MSCs stimulated with a single intensity of 0.1 (1xI-0.1) and induced toward the osteogenic lineage showed a 2-fold increase of the extracted Alizarin Red S compared to the corresponding control on day 14 (Figure 6B). Surprisingly, primary MSCs demonstrated a 4-to-5-fold increase of the extracted Alizarin Red S when stimulated with repeated shock waves of intensity 0.1 (3xI-0.1) and single shock waves of intensity 1 (1xI-1) and induced toward the osteogenic lineage compared to the corresponding control on day 14 (Figure 6B). Moreover, repeated SWA of intensity 0.1 (3xI-0.1) on MSCs indicated a 2-fold increase of the extracted Alizarin Red S compared to a single session with intensity 1 (1xI-0.1) (Figure 6B). Unexpectedly, repeated shock wave sessions of intensity 1 (1xI-1) on MSCs demonstrated a 5-fold increase of the extracted Alizarin Red S, similar to the corresponding control, which was induced toward the osteogenic lineage, on day 14 (Figure 6B).

In contrast to MSCs, the osteogenic lineage-induced MG63 cells showed no significant difference in their mineralization potential when stimulated with single and repeated shock wave sessions compared to the corresponding control (Figure 6D). Interestingly, non-induced and shock wave-stimulated MG63 cells indicated a significant increase in their mineralization potential when intensities 0.1 (1xI-0.1), 1 (1xI-1), and (3xI-1) were applied compared to the corresponding control (Figure 6D).

### Osteoblast gene marker expression after repeated shock wave application

The osteoblastic differentiation of primary MSCs and the cell line MG63 after repeated SWA was assessed using RT-PCR by investigating the relative mRNA expression of COL1A1, OCN, and RUNX2.

**FIGURE 7:**
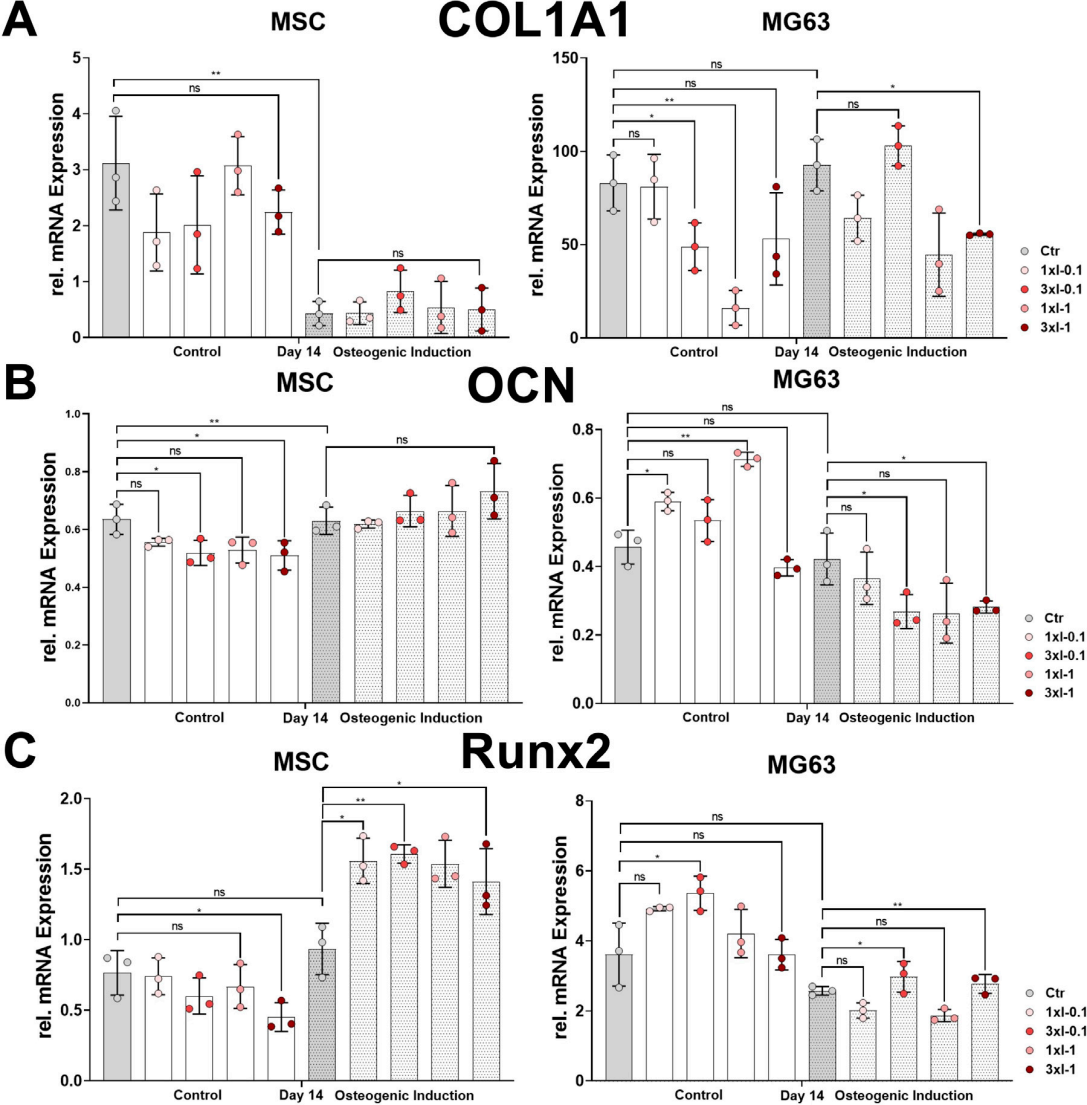
Osteoblastic gene marker expression after repeated SWA. Primary MSCs and the cell line MG63 were stimulated with repeated intensities of 0.1 (3xI-0.1) and 1 (3xI-1) and single intensities of 0.1 (1xI-0.1) and 1 (1xI-1) SWA sessions for 10 min (600 pulses) as indicated. MSCs and MG63 cells were then induced toward the osteogenic lineage for 14 days. A culture medium without any osteogenic induction supplement was used as the control. The relative mRNA expression of **(A)** COL1A1, **(B)** OCN, and **(C)** RUNX2 was investigated during the osteoblastic differentiation. Data analysis was performed using ddCT values normalized to GAPDH. Data are expressed as the average ± SD of three to five MSC donors and three to four biological replicates per group in parallel to the cell line MG63. ns: non-significant. *p < 0.05, **p < 0.01, and ***p < 0.001, two-tailed Student’s *t*-test.

The osteoblast lineage-specific gene, COL1A1, showed a decreased expression during the osteoblastic differentiation of MSCs under all conditions after repeated shock wave sessions on day 14 compared to the corresponding controls (Figure 7A). In contrast to MSCs, the relative expression of COL1A1 showed no difference between the non-induced and osteoblast-induced MG63 cell line but rather decreased after repeated SWA compared to the corresponding controls (Figure 7A). The specifically expressed gene by osteoblasts, OCN, was significantly upregulated in the osteoblastic differentiated MSCs under all conditions after repeated shock wave sessions on day 14 during the osteoblastic differentiation compared to the corresponding controls (Figure 7B). In contrast to MSCs, OCN relative expression in the cell line MG63 showed a sustained but not significant difference between non-induced and osteoblastic lineage-induced controls (Figure 7B). Interestingly, the cell line MG63 indicated a significant OCN increase after SWA of intensities 0.1 and 1 and no significant difference after repeated intensities of 0.1 (3xI-0.1) and 1 (3xI-1) compared to the corresponding control under non-induced conditions (Figure 7B). In contrast to under non-induced conditions, OCN in the cell line MG63 showed a significant decrease after repeated SWA of the intensities of 0.1 (3xI-0.1) and 1 (3xI-1), but not single intensities of 0.1 and 1, when induced toward osteoblastic differentiation compared to the corresponding control (Figure 7B). Furthermore, RUNX2 expression showed no significant difference within non-induced MSCs under all conditions after repeated shock wave sessions. Even though non-induced and osteoblast-induced control indicated no significant difference in RUNX2 expression, induced MSCs demonstrated a significant increase compared to the corresponding control under all conditions (Figure 7C). Interestingly, RUNX2 expression in the induced cell line MG63 showed a significant increase after repeated shock wave sessions of the intensities of 0.1 (3xI-0.1) and 1 (3xI-1) compared to the corresponding control, whereas non-induced cell line MG63 indicated no significant difference compared to the corresponding control except when repeated intensity of 0.1 (3xI-0.1) was applied (Figure 7C).

## Discussion

ESWT offers a non-invasive treatment option for various pathological musculoskeletal conditions that typically do not respond well to surgical interventions. The treatment with ESWT has become widely accepted, and advances in both human and veterinary medical fields have been reported (Cheng and Wang, 2015; Alvarez, 2022). Musculoskeletal pathological conditions that can benefit from ESWT include a wide range of disorders, from bone healing to tendinopathies, osteoarthritis, and osteonecrosis of the hip and cartilage (Cheng and Wang, 2015; Alvarez, 2022). Several investigations have reported the positive effects of ESWT on fracture healing and articular cartilage (Wang et al., 2003). It has been suggested that ESWT acts through a mechanotransduction mechanism to induce the reaction of bone tissue. Moreover, it has been presumed that ESWT provokes micro-fractures that consequently lead to hematoma formation resulting in callus formation and eventual fracture recovery (Hsu et al., 2003; van der Worp et al., 2013; Cheng and Wang, 2015). Nevertheless, other reported studies showed that ESWT explicitly promotes bone healing after fracture of the femur in rabbits (Wang et al., 2008). Although positive effects of ESWT in different skeletal and non-skeletal tissues were reported, clinical results also indicated some differences (Alvarez, 2022). Attention must be paid to avoid any contraindication of ESWT, such as malignant tissues, brain and spinal cord injuries, and any patient-specific condition (Cheng and Wang, 2015). To date, the working mechanism of ESWT in bone healing has not been fully understood, and therefore, basic research using MSC models *in vitro* is needed to get an insight into the underlying principles of ESWT. It has been evidenced that cells respond to a wide range of internal and external mechanical stimuli. Moreover, mechanotransduction processes have been involved in mesenchymal stem cell differentiation (Wang et al., 2009) and, therefore, represent high therapeutic potential for tissue engineering and regeneration (Martino et al., 2018).

### Optimization of shock wave generation and application setup

In our current approach, a commercial shock wave application device, PiezoWave2 (Richard Wolf GmbH, Germany), was used. Additionally, a special setup was formed to precisely expose cells to shock waves under optimal, reproducible, and well-determined conditions, like the cell number and temperature (Figure 1). However, sound field parameters such as the EFD measured in water are not directly transferable to our setup as the cell tube attenuates the shock wave. We compared the primary MSCs and the cell line MG63 through standardized cell culture processing and systematic shock wave application of single and repeated shock wave application sessions (Table 1). The current basic technical setup is easy to handle, and the achieved experimental results are reproducible and reliable.

Several reported studies have applied shock waves to cultured cells using a 2D monolayer in flasks (Raabe et al., 2013; Pemberton et al., 2015; Berebichez-Fridman et al., 2017), whereas in our approach, we applied shock waves to a 3D pellet structure of primary MSCs and the MG63 cell line. In our opinion, a 3D pellet cluster of cells is closer to the *in vivo* situation than adherent 2D monolayer cultured cells. Therefore, 3D pellet clustered cells offer the possibility to use the cells for different assays and differentiation toward different lineages, like osteogenesis and chondrogenesis, directly after SWA, without trypsinization, which might cause additional stress to the cells. Moreover, the number of cultured cells as a 2D monolayer could be a limiting factor compared to 3D pellet cells, where a huge number of cells is needed, when taking into consideration cell therapy application. Furthermore, application of shock waves on a relatively small zone for a large number of cells using 3D pellets is advantageous compared to cells cultured as 2D monolayer cells, which occupy a large surface, considering their application for cell therapy. In contrast to previous studies that investigated a limited number of parameters (Raabe et al., 2013; Salcedo-Jiménez et al., 2020), the current study systematically investigated a wide range of conditions, including the intensities and pulse numbers for single or repeated sessions of SWA (Table 1).

### Viability and proliferation of primary MSCs and the cell line MG63 after SWA

Through quantitative estimation, we determined the number of viable primary MSCs and the cell line MG63 after SWA using neutral red uptake, which is more sensitive and one of the most used cytotoxicity tests (Repetto et al., 2008).

The quantitative estimation of cell viability is based on the ability of viable cells to incorporate and bind the supravital dye neutral red in the lysosomes (Repetto et al., 2008). The dynamic and heterogenous lysosomal system is an important regulator of cellular physiology. The lysosomal distribution pattern and subcellular positioning within cells vary in response to stimuli and insults (Lakpa et al., 2021). The increase in cell viability might be due to lysosome activation after SWA and, therefore, more binding of the neutral red dye (Figure 2).

The quantitatively estimated relative viability of primary MSCs and the cell line MG63 after SWA reported in the current study was significantly affected, indicating a decreased viability with an increase in EFD applied at intensities 10, 15, and 20 (Figure 2). It has recently been reported that the relative viability of MSCs was preserved even after high-magnitude frequency was utilized (Ambattu et al., 2022). In that study, the mechanostimulation was applied on 2D monolayer MSC cultures, and their relative viability was investigated immediately without passaging them. In contrast to the reported study (Ambattu et al., 2022), we applied shock waves on MSC 3D pellets, which might be more sensitive than 2D monolayer cultures. One reason for the decreased viability at high intensities could also be the cavitation effects caused by the surrounding liquid medium of the pellets, which is not comparable to *in vivo* treatment. When comparing shock wave application *in vitro* with the *in vivo* extracorporeal shock wave treatment, the frequency-dependent attenuation by the tissue, the reflection and refraction at the tissue interfaces, and the different cavitation behavior must be taken into account. The obtained relative proliferation results for both cell types, primary MSCs and MG63, indicate a decreasing proliferation tendency on day 1 and day 3 with an increase in EFDs applied, reaching a recovery toward day 6, which is a critical time point for the osteogenic differentiation commitment of MSCs (Figure 3). We reported previously that during the differentiation process, MSCs shift toward the osteogenic commitment around day 7 compared to the controls (Haddouti et al., 2020a; Haddouti et al., 2020b). Moreover, a study on equine MSCs has reported that SWA has a relevant effect on their proliferation (Raabe et al., 2013). In contrast to the results obtained in the current study, another study reported that shock waves increased the proliferation of MG63 cells (Muzio et al., 2010). The use of different approaches, MSCs from different sources, and shock waves generated by different devices may partly explain the differences in the reported findings.

### Cytokine secretion after SWA

Most recent studies on the effects of SWA on MSCs *in vitro* did not investigate the effects of SWA on inflammatory cytokines (Raabe et al., 2013; Rohringer et al., 2014; Cat et al., 2017; Ambattu et al., 2022). A well-controlled local inflammatory microenvironment is required for effective bone regeneration. However, the signaling pathways triggered by TNFα and IL-1β that regulate bone regeneration are not fully understood (Mo et al., 2022). Recently, it has been reported that the concentrations of both proinflammatory and anti-inflammatory cytokines were found to be altered depending on the osteoarthritis stage and activity (Molnar et al., 2021). Although ESWT has been recommended for managing pain in patients suffering from knee osteoarthritis, the difference in therapeutic effects remains controversial (Liao et al., 2022). During the process of bone healing, MSCs and inflammatory cytokines interact with each other, thereby promoting the process of bone regeneration (Liu et al., 2017). In the current study, we investigated the proinflammatory cytokines IL-1β, TNFα, and IL-6, which are involved in the regulation of the proliferation and osteogenic differentiation of MSCs (Czekanska et al., 2014; Liu et al., 2017; Mo et al., 2022). It has been shown that IL-1β strongly promoted the secretion of a wide range of proteins with chemotactic, proinflammatory, and angiogenic properties, suggesting that it may have a greater influence in the early bone repair environment (Czekanska et al., 2014). In the current study, primary MSCs demonstrated a significant increase in relative secreted cytokine IL-6 when 100 and 500 pulses of intensity 5 were applied. In contrast, no significant difference in the relative IL-6 was observed at intensities 10, 15, and 20 at all number of pulses applied compared to their corresponding controls (Figure 4A). Surprisingly, the amount of relative IL-6 secreted by the cell line MG63 after the intensity 10 application was significantly higher at all pulse numbers applied compared to the corresponding control (Figure 4B). Interestingly, after application of intensities 15 and 20, the cell line MG63 showed gradually and significantly decreasing levels of the relative secreted level of IL-6. Moreover, the relative IL-6 secreted was not detected at 750 and 1,000 pulses when intensities 15 and 20 were applied (Figure 4B). Additionally, IL-1β and TNFα were not detectable in both cell types. It has been shown that shock waves cause initial inhibition of IL-6 and TNFα expression, followed by a dose-dependent enhancement of their expression in human periodontal ligament fibroblasts (Cai et al., 2016). Furthermore, it has been shown that IL-6 maintains the proliferative and undifferentiated state of MSCs, which is a critical parameter for the optimal handling of MSCs both *in vitro* and *in vivo* (Pricola et al., 2009).

### Osteogenic differentiation after single and repeated SWA

Human primary MSCs and the osteoblastic cell line MG63 were used as cell culture models to investigate the effects of SWA on osteogenic differentiation *in vitro*. We have previously shown (Haddouti et al., 2020a) that primary human MSCs from different sources fulfill the minimal criteria for defining multipotent MSCs according to the International Society for Cellular Therapy (Dominici et al., 2006). The human osteoblastic cell line MG63 is one of the most popular cell lines in osteogenesis studies (Staehlke et al., 2019). The MG63 cell line was shown to possess stable characteristics and can be considered a suitable *in vitro* model (Staehlke et al., 2019). Recently, MG63 was used as a model for trilineage differentiation in parallel to primary MSCs (Smith et al., 2023). Interestingly, it has been reported that ESWT treatment triggers both the recruitment of MSCs and their osteogenic differentiation *in vivo* (Chen et al., 2004). Nevertheless, in the current study, the osteogenic differentiation of primary MSCs and the cell line MG63 was investigated under a wide range of parameters at different time intervals, but no significant increase of the mineralization potential could be found after a single SWA compared to the corresponding controls (Figure 5). The evaluation of the osteogenic differentiation was validated via Alizarin Red S specific staining both histologically and through the optical density measurement of the extracted Alizarin Red S. Furthermore, a rather decreased mineralization potential was also observed when intensities 5 and 15 were applied on the MG63 cell line (Figure 5B). The effects of SWA on osteoblast activity have lately been reported where osteogenic gene marker expression has been investigated. The findings reported in that study indicated an inhibition of the osteogenesis manifested in a significant reduction of the typical osteogenic marker (Notarnicola et al., 2012). However, the osteogenic gene marker expressions were investigated at 24, 48, and 72 h after SWA, which may not be the optimal time point to address this issue (Notarnicola et al., 2012). Another study has reported that SWA enhanced the osteogenic medium-induced differentiation of adipose-derived stem cells into osteoblast-like cells where the osteogenic potential was evaluated through Alizarin Red S on day 28 of the osteogenic induction (Cat et al., 2017). In our opinion, both too early or too late read out of the mineralization potential during osteogenic differentiation of MSCs may overlook or miss the exact effects of the SWA. We reported previously that during the differentiation process, MSCs shift toward the osteogenic commitment around day 7 compared to the controls, and full mineralization of the extracellular matrix was reduced to 21 days instead of 28 days (Haddouti et al., 2020a; Haddouti et al., 2020b). Interestingly, it has been reported that cell proliferation and differentiation are well coordinated and show a remarkable inverse relationship (Ruijtenberg and van den Heuvel, 2016). The results of the relative proliferation on day 6 (Figure 3), corresponding in general to the stationary phase of cell growth, coincide with the osteogenic commitment during the osteogenic differentiation process. In the current study, we examined the mineralization potential during the osteogenic differentiation process at different time intervals, namely, day 3, day 7, day 14, and day 21.

Taking into consideration that there is little benefit in applying single shock wave sessions under the aforementioned working conditions to improve the mineralization potential of the primary MSCs and the line MG63, we considered applying repeated shock wave sessions over three consecutive days for 10 min corresponding to 600 pulses at intensities 0.1 and 1 and keeping the frequency constant at 1 Hz. Surprisingly, primary MSCs demonstrated a 4- to 5-fold increase in the extracted Alizarin Red S when stimulated with repeated shock wave sessions of intensity 0.1 (3xI-0.1) and single shock wave sessions of intensity 1 (1xI-1) and induced toward the osteogenic lineage compared to the corresponding control on day 14 (Figure 6B). In contrast to MSCs, the osteogenic lineage-induced MG63 cells showed no significant difference in their mineralization potential when stimulated with single and repeated SWA compared to the corresponding control (Figure 6D). Interestingly, non-induced and shock wave-stimulated MG63 cells indicated a significant increase in their mineralization potential when intensities 0.1 (1xI-0.1), 1 (1xI-1), and (3xI-1) were applied compared to the corresponding control (Figure 6D).

Furthermore, we assessed the osteoblastic differentiation of primary MSCs and the cell line MG63 after repeated SWA by monitoring the gene expression of osteoblastic markers, such as COL1A1 and RUNX2, as well as OCN, utilizing RT-PCR. The determined relative mRNA expression of COL1A1 showed downregulation in MSCs induced toward the osteoblastic lineage under all conditions compared to the corresponding controls (Figure 7A). We and others have previously reported that COL1A1 gene expression is downregulated during the osteoblastic differentiation of MSCs (Kan et al., 2014; Loebel et al., 2017; Haddouti et al., 2020b). Osteocalcin (OCN) is specifically produced by osteoblasts and is the most abundant non-collagenous protein in bone. The expression of OCN is regulated by RUNX2, which plays a major role in osteoblast differentiation and bone formation, and was shown to be expressed at a relatively similar level during *in vitro* differentiation of primary human osteoblasts (Shui et al., 2003; Zhang et al., 2011; Komori, 2020). In accordance with the previously reported findings, the expression of OCN and RUNX2 was found to be increased during osteoblastic differentiation of MSCs (Figures 7B, C). Remarkably, osteoblast-induced MSCs showed no significant difference in the expression of COL1A1 and OCN but a significant increase in RUNX2 expression after repeated SWA compared to their corresponding controls (Figure 7). In contrast to our findings, it has been reported that high-frequency stimulation triggered a significant upregulation of COL1A1 and RUNX2 and sustained OCN expression in osteoblastic differentiating MSCs (Ambattu et al., 2022).

Interestingly, osteoblast-induced cell line MG63 indicated a significant decrease in OCN expression and a significant increase in RUNX2 after repeated SWA of intensities 0.1 (3xI-0.1) and 1 (3xI-1) compared to their corresponding controls (Figures 7B, C).

In a recently reported study, bulk waves had been generated within a culture vessel, which were transmitted through the liquid or hydrogel usually with frequencies from a few hundred KHz to 40 MHz (Guex et al., 2021). The traveling bulk wave is reflected by reaching a reflector, for example, glass, and generates a standing wave (Guex et al., 2021). It has been shown that the surface acoustic wave enhanced the metabolic activity and the osteogenic differentiation of adipose tissue-derived MSCs *in vitro* (Martinez Villegas et al., 2022). Furthermore, shock waves have been reported to have beneficial effects by stimulating angiogenesis (Huang et al., 2017) and directing inflammatory responses (Basoli et al., 2020). A recent study has reported that rapid mechanical stimulation for 10 min over 5 consecutive days at high frequency (10 MHz) was shown to trigger significant upregulation in early osteogenic gene markers (Ambattu et al., 2022). The aforementioned study has reported that the mineralization potential had reached its peak after 5 days, which is unusual, as classically 28 days are reported in the literature (Cat et al., 2017), and in our previous studies, we were able to reduce the mineralization potential of MSCs from different species and sources to 21 days (Haddouti et al., 2020a; Haddouti et al., 2020b). Even though in the clinic, the treatment with ESWT is carried out during repeated sessions, standardized studies on repeated shock waves *in vitro* are barely available to date. It is of high relevance to investigate the repeated application of shock waves using an *in vitro* cell culture model in order to understand the underlying mechanism of action and the benefits and/or harm of single and repeated SWA.

In summary, MSCs are the progenitors of bone cells with the ability to differentiate into osteoblasts and then into mature osteocytes in the bone tissue (Kim and Adachi, 2021). Little is known about the basic mechanism of action of SWA on MSCs *in vitro* to date. It has been suggested that shock waves act through a mechanotransduction mechanism by inducing bone tissue reaction (Hsu et al., 2003). Moreover, a recent report introduced the growth factors induced by shock waves during osteogenic differentiation of MSCs to be involved in the process of sensing and responding to the biological effects of shock waves (Lv et al., 2023). The current approach, which is based on a specifically designed technical setup, MSCs differentiating toward the osteogenic lineage together with a systematic testing of conditions for SWA, offers a solid working model to address this question in the future. Standardizing the applied methods and conditions is of high importance for understanding the mechanism of SWA on MSCs *in vitro*, and it will improve ESWT treatment in the clinic.

## Conclusion

MSCs are a promising cell population that provides new approaches to regenerate bone tissue. ESWT is a non-invasive treatment option for many pathological musculoskeletal conditions. Studies on the effects of SWA on MSCs *in vitro* are barely available and not standardized. In the current study, a special technical setup was designed to apply shock waves on primary MSCs and the osteoblastic cell line MG63, analyze the resulting cellular responses, and eventually improve the extracorporeal shock wave treatment in the clinic. Currently, no significant difference in the osteogenic differentiation was observed at the different time intervals in both cell types after a single SWA *in vitro*. However, repeated sessions of SWA over three consecutive days using intensities 0.1 and 1 showed significant osteogenic differentiation of 4-fold or higher on MSCs at day 14, whereas no significant osteogenic differentiation was observed for the osteoblastic cell line MG63 compared to their corresponding controls. Additionally, repeated SWA was shown to trigger a significant downregulation of COL1A1, upregulation of RUNX2, and sustained increase of OCN in primary MSCs but not in the cell line MG63 when induced toward osteogenic differentiation.

We were able to determine the conditions of SWA that enhance the osteogenic differentiation of MSCs *in vitro*, therefore advancing the therapeutic potential of MSCs in organ and tissue regeneration.

## Data Availability

The raw data supporting the conclusion of this article will be made available by the authors, without undue reservation.

## Supplementary material

Supplementary Figure S1 Quantitative estimation of cell viability, relative proliferation and relative IL-6 secretion after repeated shock wave application. Primary MSCs and the cell line MG63 were stimulated with repeated intensities of 0.1 (3xI-0.1) and 1 (3xI-1) and single intensities of 0.1 (1xI-0.1) and 1 (1xI-1) shock waves application sessions for 10 min (600 pulses) as indicated. Primary MSCs and the cell line MG63 without shock wave application were used as controls (Ctr). Viable cells of both cell types normalized to the corresponding controls (%) were estimated by measuring the neutral red uptake assay on day 1 after shock waves application sessions at OD 540 nm **(A)**. The relative proliferation of both cell types normalized to the corresponding controls (%) was estimated by measuring the OD 570 nm at the indicated time intervals (day 1, 3, 6) using the MTT cell proliferation assay **(B)**. The relative IL-6 secreted was measured with Enzyme-Linked Immuno-Sorbent Assay (ELISA) normalized to the corresponding controls (%). LPS added to the control cells at a concentration of 10 µg/mL served as positive controls **(C)**. Data are expressed as average ± SD of three to five BMSC donors and four to eight biological replicates per group. Significance presented as **p* < 0.05, ***p* < 0.01, ****p* < 0.001, two-tailed Student’s t-test. * Represent significance compared to control. ns: non-significant.
