# Supplementary figures and images for: Effects of single and repeated shock wave application on the osteogenic differentiation potential of human primary mesenchymal stromal cells and the osteoblastic cell line MG63 in vitro
Source: Front Bioeng Biotechnol. 2023 Oct 12;11:1207655. doi: 10.3389/fbioe.2023.1207655 (PMC10602737; doi:10.3389/fbioe.2023.1207655)

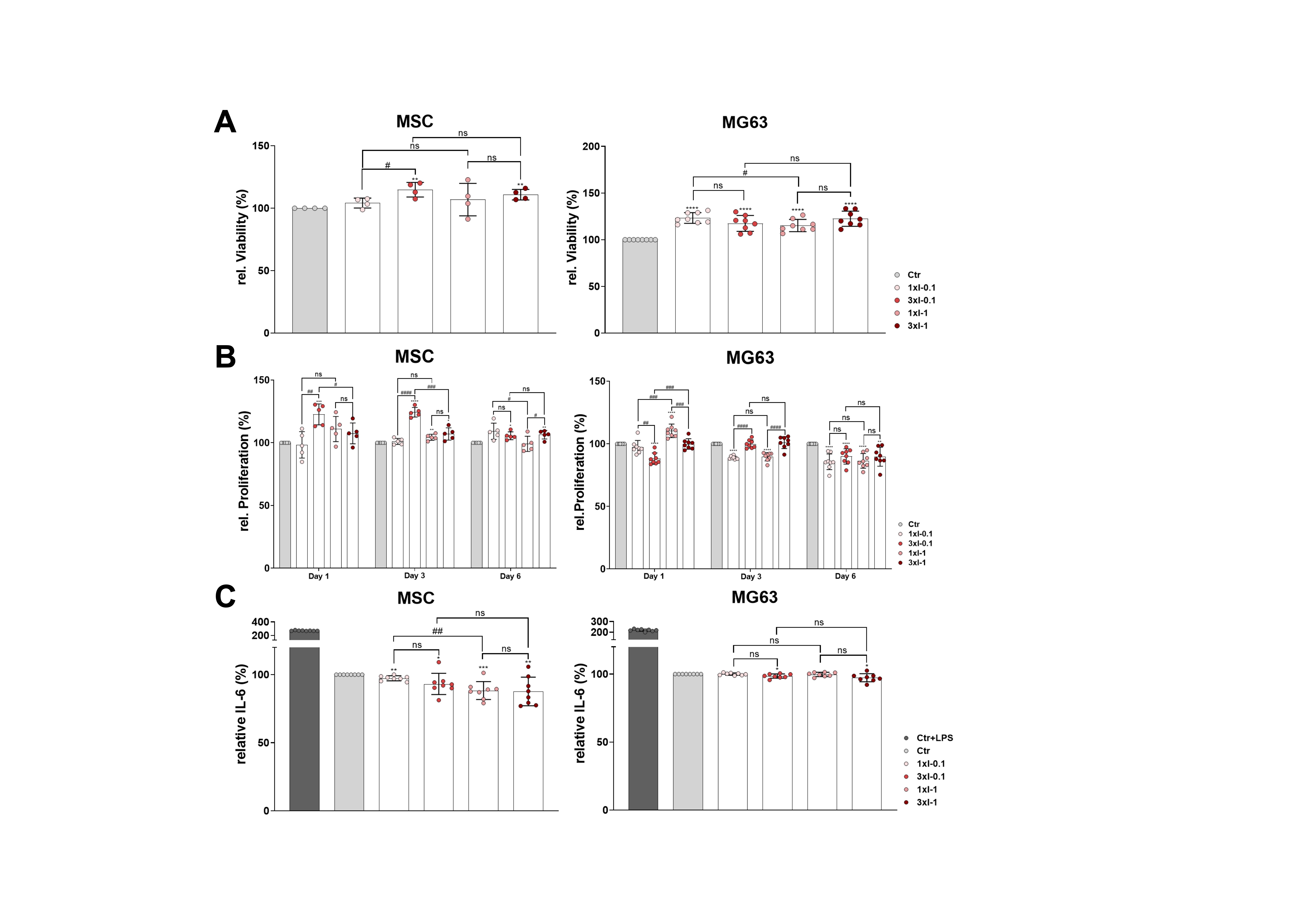

Supplement: Supplementary file 1 [file Image1.jpg]
